# Setting Priorities in Diarrhoeal Disease Research: Merits and Pitfalls of Expert Opinion

**DOI:** 10.3329/jhpn.v27i3.3372

**Published:** 2009-06

**Authors:** Wolf-Peter Schmidt

**Affiliations:** Department of Infectious and Tropical Diseases, London School of Hygiene & Tropical Medicine, Keppel Street, London WC1E 7HT, UK

In this issue of the Journal, Kosek and colleagues report the results of an exercise conducted by leading experts in diarrhoea, ranking different topics in diarrhoea research according to their potential to contribute to a substantial reduction in diarrhoeal disease by 2015 in accordance with the Millennium Development Goals (MDGs) (1). It is always easy to criticize the outcome of such expert panels. Inevitably, some researchers may feel that the merits of their particular research field were not sufficiently appreciated. It can be argued, however, that such exercises are useful if the results are interpreted appropriately.

In summing up the scores allocated to different research options, the authors found that priority was given predominantly to medical interventions and to health services research aiming at scaling up their delivery. Efficacy trials and health services research relating to rotavirus vaccination, zinc supplementation, and low-osmolarity oral rehydration solutions consistently achieved the highest scores.

As the authors noted, it came a bit as a surprise that research on nutrition (other than micronutrients) and on water, sanitation, and hygiene received much more modest scores. However, there is some logic behind this assessment, given that the experts were constrained to judging the merit of a particular research topic with regard to its potential to make a measurable contribution to reducing the burden of diarrhoea, in particular mortality, by 2015 (the deadline for the MDGs). Nutrition, water, sanitation, and hygiene are all rather complex research fields comprising numerous behavioural, political and economic aspects, which make them unlikely candidates for contributing substantially to reduction in diarrhoea by 2015. Instead, the experts recommend prioritizing clinical solutions, with the potential for a quick impact, especially on case-fatality. The question, therefore, arises whether it was legitimate in the first place to assess the different research options based on their potential to make an impact by 2015. This deadline must have made it difficult for the assessors to attribute any merit to complex interventions, for which scaling up has been found an ongoing challenge. Because some interventions require more time to develop than others, or by their nature cannot be tested in a double-blind randomized trial, it does not mean that these are less important in the longer term, nor necessarily that we can afford delaying further research at this stage.

Food, water, and sanitation are perhaps the three most important determinants of future human welfare. They are deeply affected by some of our greatest global challenges, such as the over-exploitation of land and resources, population growth, and climate change. It seems difficult to deny the merit of allocating more research efforts on how to bring these essential components of health and sustainable development to those with limited access to them. On these grounds, it can be argued that any deadline set by the international community to reach a certain development goal should only influence the speed at which interventions recognized as essential are implemented. The choice *between* different interventions may be better based on a longer-term potential, since the world (hopefully) will not come to a sudden end in 2015. Further, it is possible that, for example, research on rotavirus vaccines 10 years ago would not have been given top priority if a similar exercise for an equally short timeframe had been conducted. Today, however, rotavirus vaccines are regarded as one of the most promising tools in diarrhoea control (although their effect on all-cause mortality in low-income settings has not yet been demonstrated). Thus, the 2015 deadline might not only discourage the prioritizing of complex interventions but may discourage innovation altogether.

Kosek and colleagues suggest that the 2015 deadline may have particularly favoured health services research options, especially those on scaling up micronutrient supplementation and oral rehydration solutions. One may wonder at this point why so many health services research options were included in the exercise in the first place. As important as health services research on individual interventions is, it could be balanced with research on how to strengthen health services for diarrhoea control in more general terms, independent of a particular type of intervention that may fall out of fashion a few years down the line. A cynic might suggest that the sheer number of individual health services research topics included may have increased the likelihood of finding some of them in the top ranks.

Another factor that may have favoured clinical research options is the choice of the five criteria according to which the different research options were assessed: (a) The likelihood that the research option can yield new knowledge; (b) The likelihood that the research findings will lead to effective interventions; (c) The likelihood that the intervention derived from the research would be affordable and deliverable; (d) The most likely maximum burden of disease reduction; and (e) Impact on equity. It might have been worthwhile to include the following two further criteria, due to their importance when it comes to making policy decisions (2): (a) The likelihood that an intervention causes adverse effects and (b) The potential for an intervention to deliver non-health or indirect health benefits. Clinical approaches to diarrhoea control do not look very good in this regard. Although the risks of adverse effects may not be very large, there is always the risk that, for example, supplementation of micronutrients is only beneficial to certain populations and detrimental to others, for example, when given as a prophylaxis (3). Further, clinical solutions are rarely associated with benefits other than treating or preventing diarrhoea. This contrasts, for example, with research on scaling up school sanitation in low-income settings. School sanitation is not expected to be associated with any adverse effects but, in addition to contributing to the control of diarrhoea, helminths, and trachoma, it encourages school attendance, especially of girls, and may in the longer term increase demand for sanitation in the community as a whole (4). Should these aspects not be taken into consideration in the process of allocating research priorities?

The experts chosen for this exercise were individuals with impressive track records of research on many different public-health and clinical aspects of diarrhoea. Most of them may be regarded as generalists well-placed to judge on the wide range of different research options. However, one may question whether it was necessary to include almost exclusively medical doctors in the expert panel, leaving out professions, such as nutritional science, engineering, health policy, or environmental health. As a consequence of its composition, the panel may have been over-optimistic in judging the likelihood of scaling up medical interventions by 2015 through enhanced health services research. Many medical interventions have been around for decades and still do not reach those who need them most (5). Improving health services may be as complex as scaling up sanitation or nutrition interventions.

It could be argued that only doctors are informed enough to judge the merit of different clinical interventions and, at the same time, provide expertise in nutrition and environmental health. However, environmental health engineers or nutritionists may be as able to assume some ‘collateral expertise’, as one might call it, in the medical domain as medical doctors assume in the field of environmental health and nutrition. It may even be much easier to interpret the findings of a double-blind randomized trial of a medical intervention than research studies on complex environmental or nutritional interventions.

Would the exercise have benefited from first establishing a multidisciplinary expert panel to come up with the most useful question (which may have excluded the 2015 deadline), then using an expert panel to come up with a balanced list of research options; further, an expert panel to decide on the most suitable criteria according to which these research options are assessed; and finally, an expert panel to discuss what kind of experts should be involved in the actual exercise? It sounds a bit technocratic, and we cannot be certain that simply increasing the total number of expert panels would have strengthened the validity of the outcome. However, inclusion of a wider range of professions from the beginning may have helped avoid bias.

The authors aimed at providing “a quantitative measure of the collective optimism that research in that area can have substantial impact prior to the year 2015.” It is easy to criticize such a claim but does this mean the outcome of the exercise has little validity, or does not provide any insights that common sense would not have dictated anyway? For all their faults, expert panels as described here can be useful or even indispensable, simply because common sense does not always prevail in allocating research funds. Ideological, political and commercial considerations, at times, seem much more appealing to decision-makers than common sense. For example, it is hard to imagine what could be achieved if the research efforts invested by the pharmaceutical industry (largely funded by public-health insurance) into the development of ‘me-too’ drugs without additional health benefit compared to the original formulation could be channelled towards something more widely useful.

On the whole, despite the caveats outlined above, the article by Kosek and colleagues provides several important insights: First, there is little doubt that health services research into scaling up proven interventions should have a prominent role in short- or long-term research funding strategies. Second, as the authors point out, inferior scores for a particular public-health intervention may even indicate that more research needs to be done to strengthen the case for the intervention. The research topics with the highest scores may, in fact, represent those that already attracted considerable research efforts and funding in the past. At the risk of over-simplifying matters, it may be worthwhile to turn the ranking list upside down in the search for neglected fields requiring more attention.

Third, the result of the exercise may encourage researchers in such neglected fields to ask why it is that their field has attracted less research funding than hoped for. Possibly, the medical field has been better at conducting high-quality trials than nutritionists or water and sanitation researchers. In these fields, far-reaching policy decisions are at times based on a largely-unacceptable evidence base, as exemplified by the widespread promotion of growth monitoring (6). There are many other reasons why medical interventions regularly attract more funding, for example, due to commercial interests, professional lobbying, a favourable public relations environment, or simply the option to double-blind interventions. However, insufficient emphasis on methodological rigour in nutrition and environmental health studies may also have contributed to this situation.

Thus, the results of this exercise offer a multitude of interpretations to inform research funders. As the authors point out, the results do not prevent others from conducting a similar exercise on diarrhoeal disease research priorities, this time accounting for long-term issues, including global resources, climatic and demographic trends. Taking a long-term perspective on diarrhoea control may even diminish the need for clinical solutions.

## ACKNOWLEDGEMENTS

The author is grateful to Dirk Mueller, Suzanne Filteau, Sandy Cairncross, and Adam Biran for comments on this editorial.

Conflict of interest:

The author, a medical doctor by training, is involved in environmental health research partly funded by Unilever Ltd. and Vestergaard Frandsen, which produce health and water treatment products for use in low-income settings.
